# Copper-mediated synthesis of temperature-responsive poly(*N*-acryloyl glycinamide) polymers: a step towards greener and simple polymerisation†

**DOI:** 10.1039/d3ra04993k

**Published:** 2023-10-04

**Authors:** Nikola Křivánková, Kerem Kaya, Wouter van der Wijngaart, Ulrica Edlund

**Affiliations:** a Fibre and Polymer Technology, School of Engineering Sciences in Chemistry, Biotechnology and Health, Royal Institute of Technology (KTH) Stockholm 100 44 Sweden edlund@kth.se; b Intelligent Systems, School of Electrical Engineering and Computer Science, Royal Institute of Technology (KTH) Stockholm 100 44 Sweden; c Digital Futures, Royal Institute of Technology (KTH) Stockholm 100 44 Sweden

## Abstract

Stimuli-responsive materials with reversible supramolecular networks controlled by a change in temperature are of interest in medicine, biomedicine and analytical chemistry. For these materials to become more impactful, the development of greener synthetic practices with more sustainable solvents, lower energy consumption and a reduction in metallic catalysts is needed. In this work, we investigate the polymerisation of *N*-acryloyl glycinamide monomer by single-electron transfer reversible-deactivation radical polymerisation and its effect on the cloud point of the resulting PNAGA polymers. We accomplished 80% conversion within 5 min in water media using a copper wire catalyst. The material exhibited a sharp upper critical solution temperature (UCST) phase transition (10–80% transition within 6 K). These results indicate that UCST-exhibiting PNAGA can be synthesized at ambient temperatures and under non-inert conditions, eliminating the cost- and energy-consuming deoxygenation step. The choice of copper wire as the catalyst allows the possibility of catalyst recycling. Furthermore, we show that the reaction is feasible in a simple vial which would facilitate upscaling.

## Abstract 2

Stimuli-responsive materials with reversible supramolecular networks controlled by a change in temperature are of interest in medicine, biomedicine and analytical chemistry.

## Graphical abstract

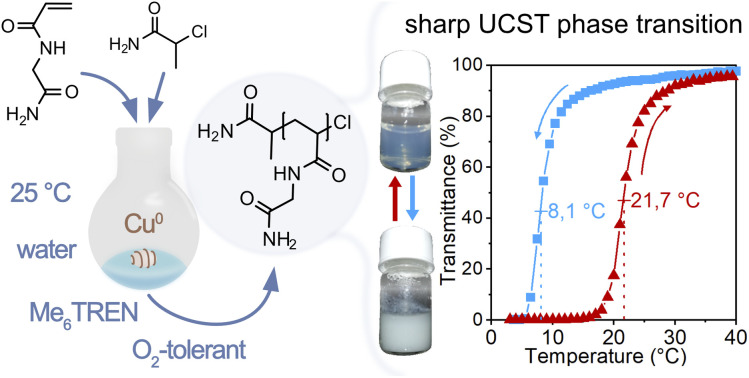

## Introduction

Poly(*N*-acryloyl glycinamide) (PNAGA) was first introduced by Haas and Schuler in 1964.^1^ This non-ionic polymer is capable of forming supramolecular networks in aqueous solutions triggered by a change in temperature. The reversible network is established with hydrogen bonds between amide groups present in the polymer side chains at temperatures below the upper critical solution temperature (UCST).

The ability of PNAGA to change properties at an external trigger has been studied for diverse applications in biomedicine, for example as a controlled drug delivery system,^2,3^ as an embolic agent^4^ or as tough and robust hydrogels.^5–8^ Furthermore, PNAGA functionalization of surfaces provides for antifouling properties^9^ or controlled cell adhesion.^10^ Li *et al.* applied PNAGA as a high-performance binder for silicon anodes.^11^ Yang *et al.* formed microgels from PNAGA for controlled catalytic activity.^12^

The sharp UCST phase transition is sensitive to the presence of ionic groups that hinder hydrogen bonding due to their strong exothermic contribution to the enthalpy of mixing. Ionic groups could be introduced unintentionally by hydrolysis of the polymer side chain, acrylate impurities in the monomer feed and improper choice of reaction conditions (*e.g.* ionic initiators or chain transfer agents).^13^ Controlled radical polymerisation (CRP) of *N*-acryloyl glycinamide (NAGA) was successfully obtained *via* reversible addition-fragmentation transfer (RAFT)^14^ and atom transfer radical polymerisation (ATRP).^15^ For RAFT polymerisation, non-ionic radical initiators and chain transfer agents were chosen to sustain the UCST transitions, however, due to the bulky nature of the end groups, the cloud points of PNAGA were dependent on the molecular weight. The study of Liu *et al.* (2013) on ATRP of NAGA with different catalytic systems and DMSO/water solutions showed PNAGA with cloud points independent of the dispersity (*Đ*) and molecular weight above 5000 g mol^−1^.^15^ They reported 80% conversion, *M̄*_*n*_ = 29 kg mol^−1^, *Đ* = 1.95 for ATRP of NAGA (DP_*n*_ = 500) in DMSO, at 45 °C, after 23 h, with the catalytic system of 2-chloropropionamide (CPA) : Me_6_TREN : CuCl : CuCl_2_ = 1 : 2 : 1 : 1. ATRP of NAGA was also studied in water, however, high *Đ* values were observed. Generally, ATRP in water poses issues, in particular, fast and uncontrolled nature caused by various side reactions altering the polymerisation rate, such as hydrolysis and elimination of the alkyl halide in initiator or polymer *ω*-end, disproportionation of Cu^I^, dissociation of the deactivating Cu^II^X_2_/L, and conventional terminating reactions.^16,17^

In 2006, Percec *et al.* introduced radical polymerisation *via* single-electron transfer.^18^ In single-electron transfer reversible-deactivation radical polymerisation (SET-RDRP), the initiator and the dormant polymer chain are suggested to be activated by Cu^0^*via* outer-sphere electron transfer (OSET) compared to the suggested inner-sphere electron transfer of Cu^I^ in ATRP.^19^ Zerovalent metals were introduced in 1997 by Matyjaszewski in Supplemental Activator and Reducing Agent (SARA) ATRP.^20^ Here, the zerovalent metal acts as a supplementary activator of the alkyl halide initiators and as a reducing agent producing activating Cu^I^*via* comproportionation of Cu^0^ with Cu^II^.^21,22^ SARA ATRP can proceed in aqueous and non-deoxygenated environments.^23–25^

SET-RDRP is a versatile technique for polymerisation of (meth)acrylates,^26^ (meth)acrylamides,^27^ vinyl chloride,^28^ or charged monomers,^29^ that can be carried out under mild conditions, without the need for strict deoxygenation, and with lower amounts of catalyst. The ppm concentrations of the catalyst ensure reduced cost and colourless reaction mixtures and render the technique more commercially exploitable compared to the synthetic procedures requiring high catalyst loadings.^30^ Cu^0^ could be introduced in different forms, such as powder,^29^ wire,^31^ tube,^32^ coin,^33^ or *in situ* formed Cu^0^ particles from the disproportionation of Cu^I^.^18^

We hypothesize that NAGA polymerisation with SET-RDRP would resolve issues encountered in the previous studies with CRP and allow synthesis with less organic solvents, metallic catalysts, and energy-demanding deoxygenation. Therefore, we studied the synthesis of PNAGA *via* SET-RDRP and explored the reaction under non-inert conditions and in water media.

For the polymerisation of PNAGA with a relatively high degree of polymerisation (DP_*n*_ = 500), the catalyst system of 2-chloropropionamide (CPA):Me_6_TREN:CuCl_2_ in the presence of a copper wire was applied. The CPA initiator has been commonly used for ATRP of NAGA.^10,15^ Furthermore, the chloride-containing initiators are considered to be more suitable for the controlled polymerisation of acrylamides compared to their bromide-containing counterparts due to easier displacement of the α-bromo chain end resulting in the loss of chain-end functionality.^34^ Tris(2-dimethylaminoethyl)amine (Me_6_TREN) is a common ligand used for ATRP as well as SET-RDRP of acrylamides.^30^ In the case of acrylamides, the *k*_p_ and *k*_t_ are generally high, therefore, we considered an externally added deactivator (CuCl_2_) to be of importance. At the beginning of the polymerisation, CuCl_2_ should ensure control over molecular weight distribution and improve the chain end fidelity of the resulting polymer.^35^ The sharp UCST phase transition of PNAGA prepared *via* SET-RDRP was confirmed.

## Experimental section

### Materials

Glycinamide hydrochloride (98%, Sigma-Aldrich), acryloyl chloride (≥96%, Sigma-Aldrich), potassium carbonate (≥99%, Sigma-Aldrich), 2-chloropropionamide (CPA, 98%, Sigma-Aldrich), copper(i) chloride (CuCl, ≥99%, Sigma-Aldrich) and tris[2-(dimethylamino)ethyl]amine (Me_6_TREN, 97%, Sigma-Aldrich) were used as received. Copper(ii) chloride (CuCl_2_, KEBO Lab) was recrystallized prior to use. Diethyl ether, acetone, dimethyl sulfoxide (DMSO), and methanol were purchased from VWR and used as received. Ultrapure water was obtained from the Milli-Q water system IQ-7000.

### Analytical methods

#### Differential scanning calorimetry (DSC)

The purity of the synthesized NAGA monomer was confirmed by the melting temperature determined by DSC (Mettler Toledo, heating rate 10 K min^−1^, under N_2_ flow 50 mL min^−1^, from 100 to 200 °C) and compared with the values in the literature.^13^ Software STARe (version 15.00b) was used for data acquisition and processing.

#### High-performance liquid chromatography (HPLC)

The purity of the monomer was further validated on HPLC (Thermo Scientific Dionex UltiMate 3000) with Rezex ROA Organic acid columns connected to a RI (Water 2414) detector. The monomer was analysed in a 2.5 mM H_2_SO_4_ eluent (4 mg mL^−1^), at 50 °C and flow 0.5 mL min^−1^. The elugram is included in the ESI (Fig. S3).†

#### Nuclear magnetic resonance (NMR)

^1^H NMR spectra were recorded on the Bruker Ultrashield™ (400 MHz) instrument in DMSO-*d*_6_ or D_2_O. NAGA monomer was analysed at room temperature whereas the polymer samples were analysed at 50 °C. The conversions were determined from spectra by comparing the peak areas from vinyl protons (6.44–6.19 ppm, 6.19–5.98 ppm, and 5.71–5.46 ppm) to the peak area of polymer backbone (2.25–1.03 ppm CH_2_ and CH) and averaged (Fig. S4†). Data analysis was performed using the software MestReNova (version 14.2.0.-26256). The obtained conversion data were fitted with a five-parameter logistic function (5PL) shown in eqn (1).1
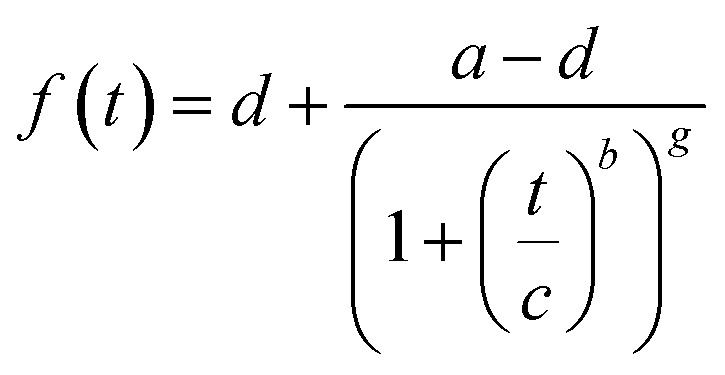


The apparent rate constant (*k*^app^_p_) was determined from the maximum of the first derivative of the curve fit.

#### Size exclusion chromatography (SEC)

The molecular weight and dispersity (*Đ* = *M̄*_w_/*M̄*_*n*_) of the synthesized polymers were determined by SEC (SECcurity 1260 GPC System, PSS) with 0.5 w/w% LiBr in DMSO as eluent. PSS Gram columns (100 Å and 10 000 Å, 300 mm length, 8.0 mm diameter and 10 μm particle size) calibrated with pullulan standards and tempered at 60 °C together with the differential refractometer detector (d*n*/d*c* = 0.089) were employed. The flow rate was set to 0.5 mL min^−1^ and the injection volume to 100 μL. Samples were dissolved in the eluent overnight at 60 °C. To prevent aggregation as much as possible, the samples were sonicated at 60 °C and filtered through a 0.45 μm Teflon filter right before injecting the sample into the SEC instrument. The time spent at lower temperatures before the analysis was reduced. The raw data were analysed using the PSS WinGPC Unichrom software. The initiator efficiency was calculated from the ratio of theoretical (from NMR) to real molecular weight obtained from the SEC analysis. The molecular weight distributions are included in the ESI.†

#### UV/vis spectrophotometry

Turbidimetry experiments were performed on UV/vis spectrophotometer (UV2550 Shimadzu) equipped with a temperature controller (S-1700) at 670 nm wavelength. Purified samples were dissolved in polypropylene tubes for two hours at 60 °C to obtain 1 wt% solutions. The cuvette and the cuvette holder were heated to 50 °C, the sample was quickly transferred to the cuvette and the measurement of the cooling curve was performed from 50 °C to 3 °C at the rate of 1 °C min^−1^ with 10 s wait time before each transmittance acquisition. The sample was kept at 3 °C for 5 min before proceeding with the heating cycle measurement from 3 to 50 °C under the same conditions as for the cooling cycle. The sample was constantly stirred during the measurement. The cloud point was determined as the inflection point of the transmittance curve.

### Synthesis of *N*-acryloyl glycinamide (NAGA, IUPAC name *N*-(2-amino-2-oxoethyl)prop-2-enamide) monomer

High-purity NAGA monomer was synthesized according to the guidelines introduced by Seuring *et al.*^13^ and purified as reported by Makinen *et al.*^36^ Glycinamide hydrochloride (3 g) and potassium carbonate (7.5 g) were dissolved in deionized water (50 mL) under stirring in an ice bath. A solution of acryloyl chloride (2 mL) and cold anhydrous diethyl ether (100 mL) was added dropwise over 30 min under vigorous stirring to the aqueous solution in the ice bath while keeping the whole apparatus in the dark. The reaction proceeded at room temperature for 2 h. Afterward, the diethyl ether phase was removed by rotary evaporation. The aqueous phase was neutralized by 5 M hydrochloric acid and freeze-dried. The crude product was extracted with acetone (6 × 90 mL, 40 °C, 15 min). The insoluble potassium salts were filtered off and a portion of acetone was removed by rotary evaporation. The obtained solution of the raw product in acetone (180 mL) was placed at −30 °C overnight. The formed crystals were filtered and re-dissolved in a methanol/acetone solution (1 : 2 v/v, 17 mL, 55 °C). The solution was placed back at −30 °C. The recrystallized product was filtered and vacuum dried. The synthesis was repeated up to 30 times.

### SET-RDRP of NAGA with copper wire

Firstly, 18-gauge copper wire was cut either into 4 or 6 cm and washed in acetone. The surface of the wire was scratched using sandpaper. The wire was wrapped around a cross-shaped stirring bar, washed in methanol, and dried under a nitrogen atmosphere before use.

**Scheme 1:**
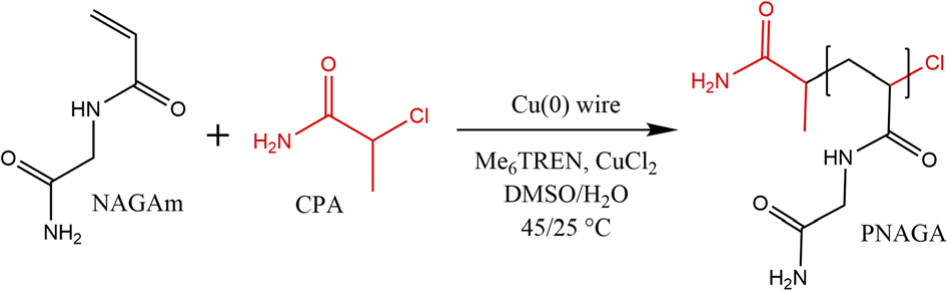
General conditions of copper wire mediated NAGAm polymerisation by single-electron transfer radical polymerisation.

In a typical reaction (Scheme 1), the monomer (NAGA, 1 g, 7.8 mmol), initiator (CPA, 1.7 mg, 0.016 mmol), ligand (Me_6_TREN, 8.3 μL, 0.031 mmol) and CuCl_2_ (2.1 mg, 0.016 mmol) were dissolved in 5.2 mL of the solvent (Milli-Q water, DMSO, or a Milli-Q water/DMSO mixture) in a 25 mL Schlenk flask. In the case of degassed polymerisation conditions, three freeze–pump–thaw cycles were performed, whereas for non-degassed samples, the degassing cycles were skipped, and the reaction was performed under a nitrogen blanket. The flask was heated to 25 °C and the reaction started by dropping the stirring bar with the copper wire in the reaction mixture. For the study of kinetics, 0.3 mL of the reaction mixture was sampled at specific intervals through a septum and dried in a vacuum oven. After the polymerisation, the stirring bar and the wire were removed and the formed polymer was precipitated in 10-fold excess of cold methanol. The product was centrifuged (8000 rpm, 10 min, three cycles) and dried in the vacuum oven for 24 h.

### SET-RDRP of NAGA *via* disproportionation of CuCl

NAGA (1 g, 7.8 mmol), CPA (1.7 mg, 0.016 mmol), and Milli-Q water (3.2 mL) were charged into a Schlenk tube and degassed with three cycles of freeze–pump–thaw. In a separate Schlenk flask, Me_6_TREN (8.3 μL, 0.031 mmol) and CuCl (3 mg, 0.03 mmol) were added to 2 mL of Milli-Q water and degassed with three freeze–pump–thaw cycles. The CuCl was let to disproportionate for 30 min. Cu^0^ particles appear at the bottom of the flask. The polymerisation starts when the aforementioned reaction mixture is transferred through a cannula into the Schlenk flask containing the *in situ* formed Cu^0^ particles. The purification procedure was kept the same as for SET-RDRP of NAGA with copper wire.

### *In situ* chain extension

The chain-extension experiments followed the steps described in the paragraph SET-RDRP of NAGA with copper wire. The polymerisation proceeded in Milli-Q water, at deoxygenated conditions (after three cycles of freeze–pump–thaw), at either 25 or 45 °C. The reaction conditions were the following: [M]_0_ : [I]_0_ : [L]_0_ : [Cu^II^]_0_ = 50 : 1 : 2 : 1, with initial monomer concentration of 1.5 M and 6 cm of a copper wire (gauge 18). The polymerisation proceeded in 2.6 mL of Milli-Q water for one hour. Afterwards, 1.3 mL of the reaction mixture was withdrawn for analysis (DP_*n*_ = 50) and 1.3 mL of deoxygenated solution of 25 eq. of NAGA in Milli-Q was added. The polymerisation was let to run for another hour to obtain the extended polymer chains with theoretical DP_*n*_ = 100.

## Results and discussion

It is crucial to start from NAGA monomers of high purity to achieve a polymer that undergoes an upper critical solution temperature (UCST) phase transition, because the presence of ionic groups and impurities in the monomer would suppress the phase transition properties.^13^ The monomers synthesized in this work exhibited a melting temperature ranging from 138.8 to 141.2 °C. The DSC thermogram and the NMR spectrum are included in the ESI (Fig. S1 and S2).† The NAGA monomer was subsequently polymerized *via* SET-RDRP under varied conditions. Firstly, the effect of solvent polarity on the kinetics at 45 °C and 25 °C was investigated. Thereafter, the kinetics without deoxygenation were studied. The next section compares the polymerisations in water with varied ratios of CuCl_2_ and ligand. Subsequently, the influence of CuCl_2_, the copper wire length, and the recyclability of the wire were determined. The paper concludes with the confirmation of the UCST phase transition properties.

### The effect of solvent polarity on SET-RDRP kinetics of NAGA at 45 °C

The initial experiments were carried out at 45 °C in either water, DMSO, or their mixture because PNAGA has been shown to dissolve only in water and DMSO^37,38^ above the cloud temperature. In previous studies, an increase in the solvent polarity resulted in accelerated polymerisation kinetics of Cu(0)-mediated SET-RDRP while maintaining the control.^27,39^ The increasing solvent polarity promotes electron transfer as well as the regeneration of Cu^0^ and Cu^II^ during the reaction.^40^ Furthermore, environmentally benign solvents should be always prioritised as stated in the ACS Green Principles laid in 1998.^41^

The experiments proceeded with CPA : Me_6_TREN : CuCl_2_ in ratio 1 : 2 : 1, monomer concentration 1.5 M and were degassed with three freeze–pump–thaw cycles. The reaction was initiated by dropping the copper wire (6 cm, scratched, washed in methanol) wrapped around the stirring bar into the reaction medium.

**Fig. 1:**
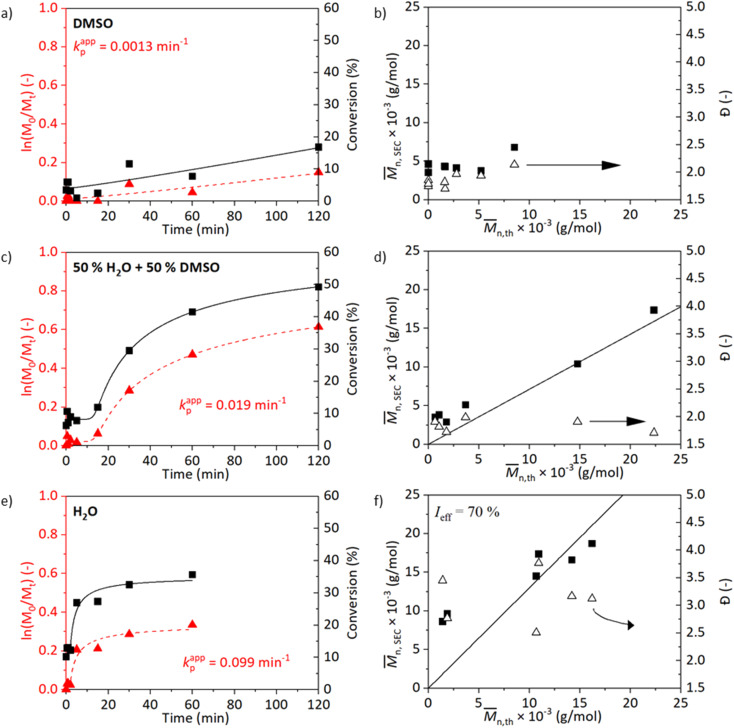
Kinetic plots and the evolution of molecular weight and dispersity for SET-RDRP of NAGA in degassed DMSO (a and b), DMSO/H_2_O (1/1, v/v, c and d) and H_2_O (e and f) at 45 °C under the conditions [M]_0_ : [I]_0_ : [L]_0_ : [Cu^II^]_0_ = 500 : 1 : 2 : 1 and catalysis of a copper wire (6 cm). Data were fitted with a 5PL fit. The *k*^app^_p_ was calculated as the maximum of the first derivative.

The kinetics in either water, DMSO, or their mixture are shown in Fig. 1. While processing the kinetics data, we could observe different stages in polymerisation – lag phase (induction time), growth phase, and plateau phase (termination). To reduce subjectiveness in determining *k*^app^_p_, we least-mean-square fitted the measurement data with a five-parameter logistic function (5PL, eqn (1)). This 5PL fit reconstitutes all three stages mentioned previously as well as deviations. The *k*^app^_p_ was calculated as the maximum of the first derivative of the curve. The comparison between the traditional and the 5PL fit is shown in Fig. S5.†

Fig. 1a and b show results of polymerisation in DMSO, Fig. 1c and d in a water/DMSO solution (1/1 v/v), and Fig. 1e and f in water. A trend in the *k*^app^_p_ can be observed from the observed conversions (Fig. 1a, c, and e): *k*^app^_p_ increases with the increasing polarity of the reaction medium. The polymerisation in DMSO evolves slowly and reaches a 17% conversion after 2 h (Fig. 1a). The molecular weight of the polymer does not linearly correlate with the theoretical molecular weight calculated from the NMR data. The conversion, as well as the controlled nature of the reaction, improves in the case of water/DMSO medium (Fig. 1c). After a short induction time (5 min), the reaction proceeds and a 45% conversion is achieved after 2 h. After 50 min, the stirring bar struggled to rotate due to the formation of a hydrogel network resulting in a decrease in the polymerisation rate. Improved evolution of molecular weight is achieved. The polymerisation carried out in water progresses fast at the beginning of the reaction resulting in a 27% conversion already after 5 min and a 36% conversion is obtained after 1 h. The fast termination observed in Fig. 1e could be caused by the loss of chain-end functionality which is promoted by the temperature and the choice of water as the medium. A shoulder in the molecular weight distribution is visible in Fig. S6† confirming this hypothesis. The presence of dead chains would also explain the high *Đ*.^42^ In the study of Liu *et al.* (2013) on ATRP of NAGA in water, a high *Đ* of 2.88 was observed when the reaction was carried out in water at 30 °C, DP_*n*_ = 200, with 2-bromopropionamide (BPA) : CuBr : CuBr_2_ : Me_6_TREN (1 : 1 : 1 : 2) as a catalyst system.^15^

### The effect of solvent polarity on SET-RDRP kinetics of NAGA at 25 °C

SET-RDRP is generally carried out at ambient and lower temperatures reducing the loss of chain-end functionality and ensuring better control over the reaction.^30^ Since we are aiming towards greener chemistry in our study, conducting the polymerisation without the need for heating was definitely of interest. The SET-RDRP of NAGA was carried out at 25 °C under the same conditions as at 45 °C ([M]_0_ : [I]_0_ : [L]_0_ : [Cu^II^]_0_ = 500 : 1 : 2 : 1, 6 cm of wire, deoxygenated with three cycles of freeze–pump–thaw) in different solvents, DMSO, DMSO/water (1/1 v/v) and water.

**Fig. 2:**
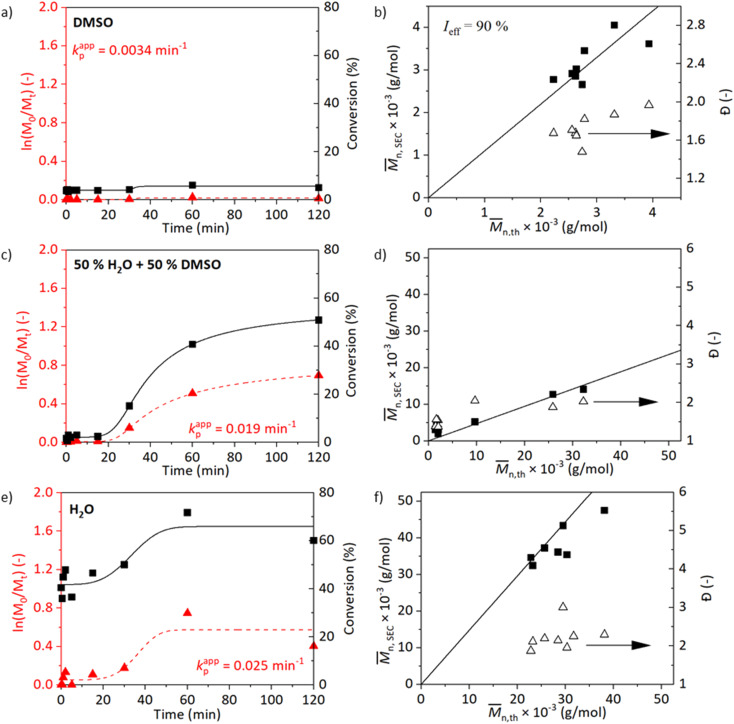
Kinetic plots with the evolution of molecular weight and dispersity for SET-RDRP of NAGAm in degassed DMSO (a and b), DMSO/H_2_O (1/1, v/v, c and d) and H_2_O (e and f) at 25 °C under the conditions [M]_0_ : [I]_0_ : [L]_0_ : [Cu^II^]_0_ = 500 : 1 : 2 : 1 and catalysis of a copper wire (6 cm). Data were fitted with a 5PL fit. The *k*^app^_p_ was calculated as the maximum of the first derivative.

The kinetics with the resulting molecular weights and *Đ* values of the reactions at 25 °C are shown in Fig. 2. Similar to the kinetics at 45 °C, the trend of increasing *k*^app^_p_ with increasing polarity of the solvent medium is observed (Fig. 2a, c, and e). The reaction in DMSO at 25 °C did not show an increase in the conversion when left running for 2 h (Fig. 2a and b). Improved conversions are observed for kinetics of experiments carried out in media containing water (Fig. 2c and d). The reaction carried out in a medium water/DMSO attained a conversion of 51% after 2 h with *Đ* = 2. The molecular weight analysed by SEC did not correlate well with the calculated value from NMR. The kinetics at 45 and 25 °C in water/DMSO medium are comparable. The reaction in water proceeded rapidly in the beginning and reached the maximum conversion of 78% after 1 h with *Đ* = 2. The observed shoulder in the molecular weight distribution of the sample synthesized in water at 45 °C disappeared when the temperature was reduced to 25 °C, however, peak tailing is still visible (Fig. S7†). Furthermore, chain-extension experiments both at 25 and 45 °C were conducted and prove the presence of the living chain ends (Table S1 and Fig. S12†).

Overall, the polymerisations occurring in media containing water proceeded at a faster rate than in pure DMSO correlating with the previously mentioned studies. However, increased *Đ* was observed as well.

### SET-RDRP without deoxygenation

Cu^0^-mediated polymerisations have been previously described as oxygen tolerant in various applications.^43^ The elemental copper serves as an effective oxygen scavenger. Generally, an induction time is observed at the beginning of the reaction, and the polymerisation proceeds once the oxygen is consumed.^44^ Therefore, a closed container for the reaction is crucial. Being able to eliminate the degassing step would be very beneficial as it is usually quite time-consuming and requires special equipment (*e.g.* Schlenk line). Furthermore, the elimination of freeze–pump–thaw pre-treatment would significantly improve the prospects of upscaling.

**Fig. 3:**
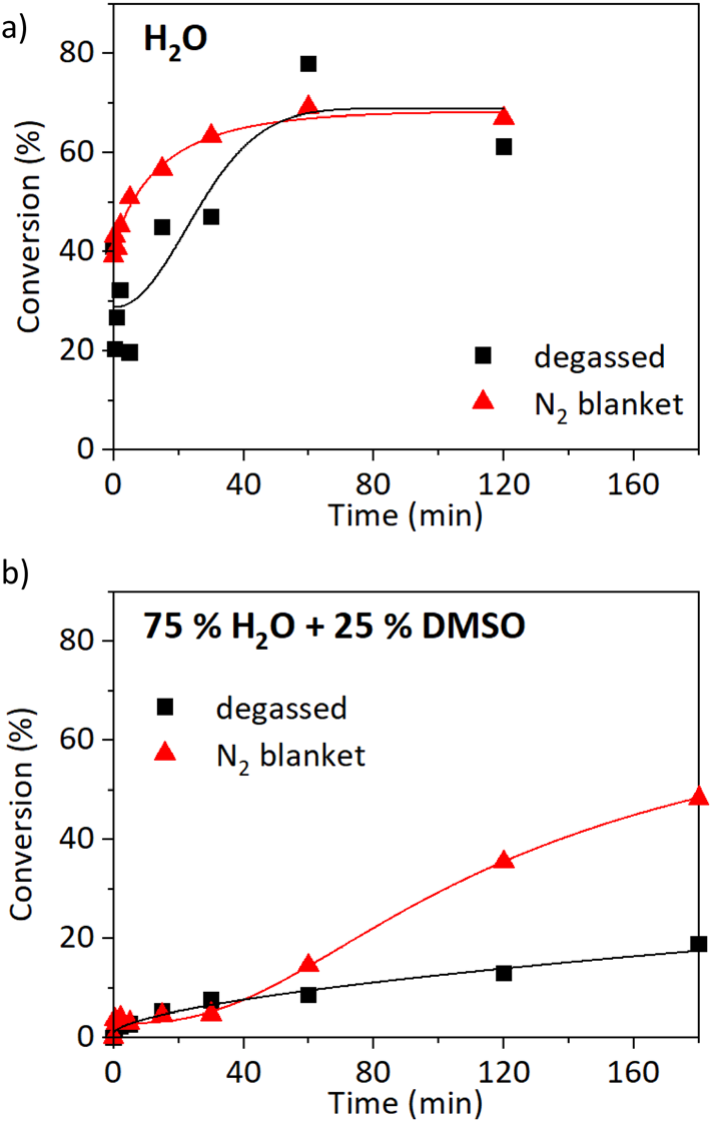
Oxygen tolerance of SET-RDRP of NAGAm at 25 °C. Comparison of conversions after three cycles of freeze–pump–thaw degassing (■) and without degassing (▲) in either water (a) or 75% water and 25% DMSO solution (b). Data were fitted with a 5PL fit.

First of all, the SET-RDRP of NAGA was carried out at 25 °C ([M]_0_ : [I]_0_ : [L]_0_ : [Cu^II^]_0_ = 500 : 1:2 : 1, 6 cm of wire) without the freeze–pump–thaw degassing. The reaction media was not even tediously purged beforehand, the polymerisation proceeded under a nitrogen blanket instead. The comparison of kinetics with and without deoxygenation in water is shown in Fig. 3a and in water/DMSO solution (3/1 v/v) in Fig. 3b. The polymerisation in water proceeded comparably with and without deoxygenation (Fig. 3a). The maximum conversion was reached after 1 h, 78% in the case of the degassed sample, and 69% for the non-deoxygenated sample. The *M̄*_*n*_ of the degassed sample was 30 700 g mol^−1^ with a high *Đ* of 3.1. The experiment without the deoxygenation step resulted in an improved *Đ* of 1.95 (*M̄*_*n*_ = 28 600 g mol^−1^, Fig. S8a†). When the reaction was carried out in a solution of 75% water and 25% DMSO, higher conversions were observed in the case of non-degassed conditions (Fig. 3b). The conversion reached 19% after 180 min (*M̄*_*n*_ = 9800 g mol^−1^, *Đ* = 2.4) when three cycles of freeze–pump–thaw were performed, compared to 48% without degassing (*M̄*_*n*_ = 28 000 g mol^−1^, *Đ* = 1.94, Fig. S8b†). We can conclude that the SET-RDRP of NAGA can be performed well also without deoxygenation at an ambient temperature.

We wanted to take it a step further and see whether PNAGA can be prepared *via* SET-RDRP also in a very common container. The polymerisation ([M]_0_ : [CPA]_0_ : [Me_6_TREN]_0_ : [CuCl_2_]_0_ = 500 : 1 : 0.2 : 0.1, 6 cm of wire, [NAGA]_0_ = 0.75 M) was carried out in a typical 10 mL borosilicate vial closed with a polyethylene snap cap at room temperature and air atmosphere. The reaction was left undisturbed for 4 h and 72% of conversion was achieved. The resulting molecular weight was 39 000 g mol^−1^, with *Đ* = 2.2 (Fig. S9†). Liarou *et al.* conducted a study previously on copper-mediated polymerisations in closed vials without prior deoxygenation of the reaction media. Their findings show that the present oxygen is not consumed only by the elemental copper, but that the initiator is prominently participating as well. Due to this, the initiator efficiency might be decreased.^45^ In our case, the deviation between the theoretical *M̄*_*n*_ and the one determined by SEC was in an acceptable range (45 700 and 39 100 g mol^−1^, respectively). Being able to conduct a SET-RDRP of PNAGA in such a simple container (glass vial) without tedious preparations simplifies incredibly the whole procedure and renders upscaling more possible.

### SET-RDRP of NAGA in water

The polymerisations in the deoxygenated conditions at 25 and 45 °C were carried out with quite increased ratios of ligand and Cu^II^ (CPA : Me_6_TREN : CuCl_2_ = 1 : 2 : 1). This ratio of Cu^II^ and Me_6_TREN is commonly applied for ATRP of NAGA.^15^ The Cu^II^ serves as a deactivator generating dormant species during the polymerisation ensuring the controlled nature of the reaction. When the reactive Cu^0^ is generated *via* disproportionation of Cu^I^, Cu^II^ forms as well. However, in the case of copper wire catalysis, externally added Cu^II^ may be crucial. As acrylamides generally exhibit high *k*_p_ and *k*_t_, the added Cu^II^ assures control in the early stages of polymerisation.^29^ Furthermore, the addition of Cu^II^ has been shown to decrease the induction time.^31^ Generally, lower ratios are needed for SET-RDRP compared to ATRP. A previous study by Jones *et al.* concluded that higher Cu^II^ and ligand ratios should be employed when aiming for polyacrylamides with increased degrees of polymerisation (DP_*n*_ > 360) to sustain the control of the polymerisation.^46^ Since our results showed quite increased *Đ* values, we wanted to explore whether the results would improve with different Cu^II^ and ligand ratios.

Table 1 shows kinetic data until the highest conversion of experiments with varying ratios of Me_6_TREN and CuCl_2_ carried out in water, at 25 °C. Entries 1 and 2 represent samples discussed already in previous sections with Me_6_TREN : CuCl_2_ ratio of 2 : 1. Only 1 is deoxygenated while the other reactions (2–6) proceeded under a nitrogen blanket. Firstly, SET-RDRP of NAGA was investigated with even higher ratios of Me_6_TREN and CuCl_2_ than before (Me_6_TREN : CuCl_2_ = 4 : 2) (3, Fig. S10†). For sample 4, we followed the suggested concentration ratio of Me_6_TREN by Nguyen *et al.*: [Me_6_TREN]_0_ = 0.1 × [I]_0_ + [CuCl_2_]_0_, while keeping the [CuCl_2_]_0_ = 1.^27^ In entries 5 and 6, the calculations of ligand were kept the same as for 4 but this time the ratio of CuCl_2_ was set to 0.1 equivalent relative to the initiator as for acrylamides the value is typically between 0.05 to 0.1.^35^ For entry 6, the reaction mixture was diluted to 0.75 M compared to 5 (1.5 M).

**Table 1:** The effect of various ligand per Cu^II^ ratios on the polydispersity and kinetics. The polymerisations occurred at 25 °C, in water, under an N_2_ blanket, with DP_*n*_ = 500, and 6 cm of copper wire

Entry	L : Cu^II^	*c*_0_ a [M]	Time [min]	Conv. [%]	*M̄*_*n*,theo_ [g mol^−1^]	*M̄*_*n*,SEC_ [g mol^−1^]	*Đ*	*k* ^app^_p_ [min^−1^]
1	2 : 1^b^	1.5	5	20	12 600	11 100	3.92	0.0102
			30	47	29 800	36 600	3.49	
			60	78	49 300	30 700	3.13	
2	2 : 1	1.5	5	51	32 300	22 500	1.98	0.0506
			30	63	40 100	20 800	2.05	
			60	69	43 700	28 700	1.95	
3	4 : 2	1.5	5	56	35 600	27 400	2.13	0.0050
			30	41	26 100	29 900	2.62	
			240	81	51 400	25 700	1.86	
4	1.1 : 1	1.5	15	9	6000	16 200	2.66	0.0327
			30	43	27 600	30 200	2.38	
			120	76	48 000	36 000	1.99	
5	0.2 : 0.1	1.5	5	34	21 400	34 200	2.02	0.2300
			30	48	30 200	41 900	1.82	
			60	57	36 200	53 200	2.49	
6	0.2 : 0.1	0.75	5	49	31 100	45 600	2.10	0.2650
			30	21	13 100	36 100	1.86	
			60	52	33 200	40 100	1.87	

a Initial monomer concentration.

b Deoxygenated by three cycles of freeze–pump–thaw.

The conditions employed in reaction 4 resulted in an improved conversion of NAGA while keeping the *Đ* similar to 2 (1.99 and 1.95, respectively). When the equivalents relative to the initiator of Cu^II^ and ligand were doubled (entry 3), maximum conversion was reached after 4 h with *Đ* = 1.86. The theoretical *M̄*_*n*_ is however significantly higher than *M̄*_*n*_ detected by SEC (51 400 and 25 700 g mol^−1^, respectively). This could be caused by premature termination or the loss of chain-end functionality of the chains. The opposite behaviour, which signifies decreased initiator efficiency, was observed in entries 5 and 6 where the equivalents of Cu^II^ and Me_6_TREN were lowered 10-fold. For entry 5, at the highest conversion (60 min), the theoretical *M̄*_*n*_ was 36 200 g mol^−1^ and the obtained *M̄*_*n*_ was 53 200 g mol^−1^. Furthermore, *Đ* increased to 2.49. The high *Đ* could be explained by slow exchange reactions or inhomogeneity of the reaction media due to the formation of a hydrogel network. The presence of a hydrogel network can be observed macroscopically due to the decrease in the stirring bar mobility. Entry 6 shows that dilution to a lower concentration of monomer improved significantly the *Đ* (1.87, Fig. S11†). To sum up, the concentration ratios of Cu^II^ and Me_6_TREN equivalent to the initiator can be considerably reduced for SET-RDRP of NAGA in water. By minimizing the amount of externally added CuCl_2_, the amount of copper salts needed to be removed during purification is reduced. However, the *Đ* is not considerably improved. Therefore, the cause of high *Đ* is probably the loss of chain-end functionality as the hydrolysis of the alkyl halide at the *ω* chain end is independent of the copper concentration and easily occurs in aqueous solutions.^16^ Furthermore, as the apparent polymerisation rate constant (*k*^app^_p_) decreases with increasing concentration of externally added CuCl_2_, we can conclude that the formation of CuCl *via* comproportionation is not favoured.^47^

### The effect of the wire area

As an elemental copper is used as a catalyst, the SET-RDRP is surface-activated and therefore the rate of polymerisation is surface dependent. The *k*^app^_p_ for polymerisation of methyl methacrylate was reported to be proportional to the 0.44th power of the surface area of wire.^48,49^

We carried out SET-RDRP of NAGA under the catalysis of copper wire with varying lengths in water, at 25 °C, under an N_2_ blanket, with [NAGA]_0_ : [CPA]_0_ : [Me_6_TREN]_0_ : [Cu^II^]_0_ = 500 : 1 : 0.2 : 0.1, [NAGA]_0_ = 0.75 M (Table 2). The maximum conversion of 52% was achieved after 60 min (*M̄*_*n*,SEC_ = 40 100 g mol^−1^, *Đ* = 1.87) when 6 cm of an 18-gauge wire was used. On the other hand, with only 4 cm of 18-gauge wire, the maximum conversion was reached already after 5 min and increased to 80%. At 5 min the *M̄*_*n*,SEC_ was determined to be 52 600 g mol^−1^ (*Đ* = 2.1) and at 30 min the *Đ* decreased to 1.92. Therefore, with the shorter (4 cm) copper wire, the SET-RDRP of NAGA reached higher conversions in a shorter time and kept comparable *Đ*. With the longer wire, the rate of activation was probably higher than that of deactivation, leading to premature termination, resulting in lower conversions.

**Table 2:** The effect of copper wire length on the SET-RDRP of NAGA in water at 25 °C and under a nitrogen blanket without degassing. Reaction conditions were [M]_0_ : [I]_0_ : [L]_0_ : [Cu^II^]_0_ = 500 : 1 : 0.2 : 0.1, [M]_0_ = 0.75 M

*l* a [cm]	Time [min]	Conv. [%]	*M̄*_*n*,theo_ [g mol^−1^]	*M̄*_*n*,SEC_ [g mol^−1^]	*Đ*	*k* ^app^_p_ [min^−1^]
6	5	49	31 100	45 600	2.10	0.2650
	30	21	13 100	36 100	1.86	
	60	52	33 200	40 100	1.87	
4	1	52	33 100	59 800	2.09	0.4666
	5	80	50 500	52 600	2.10	
	30	80	50 800	43 900	1.92	

a Length of copper wire (gauge 18).

### Different sources of Cu^0^: copper wire *vs.* disproportionation

Disproportionation of Cu^I^ was the common source of Cu^0^ in previous studies on SET-RDRP of acrylamides in water,^46,50–53^ and only a few reported the application of the copper wire.^27,54^ However, the use of copper wire facilitates a simple experimental setup and simplifies the purification of the product.

The comparison of SET-RDRP of NAGA in water catalysed by either wire or *in situ* formed Cu^0^ particles is reported in Table 3. The polymerisations were carried out under deoxygenated conditions at 25 and 45 °C. SET-RDRP of NAGA at 45 °C proceeded very fast with *in situ* formed Cu^0^ (>99% in less than 10 min). PNAGA exhibited also much lower *Đ* (1.4 compared to 3.1). At 25 °C, the conversions of the wire-catalysed SET-RDRP improved to 78% in 60 min, however, *in situ* catalysis led to a faster reaction (86% in less than 1 min). *Đ* increased in the sample polymerized at 25 °C compared to 45 °C (2.2 and 1.4, respectively) but was still lower than in the case of the wire-catalysed polymerisation.

**Table 3:** SET-RDRP of NAGA ([M]_0_ = 1.5 M) in water under deoxygenated conditions catalysed either with copper wire (6 cm) or *in situ* formed Cu^0^ particles at 25 and 45 °C

Cu^0^ source	[M]_0_ : [I]_0_ : [L]_0_ : [Cu^I^]_0_ : [Cu^II^]_0_	*T* [°C]	Time [min]	Conv. [%]	*M̄*_*n*,theo_ [g mol^−1^]	*M̄*_*n*,SEC_ [g mol^−1^]	*Đ*
Wire	500 : 1 : 2 : — : 1	45	60	36	16 200	18 700	3.1
Cu^I^	500 : 1 : 2 : 2 : —	45	<10	>99	64 000	40 900	1.4
Wire	500 : 1 : 2 : — : 1	25	60	78	49 300	30 700	3.1
Cu^I^	500 : 1 : 2 : 2 : —	25	<1	86	54 700	31 300	2.2

From our reported results, the disproportionation of CuCl yields better results compared with the copper wire when following the conditions stated in Table 3.

To demonstrate the advantage of copper wire as a catalyst - its reusability - three repetitions of SET-RDRP of NAGA were performed with the same wire and the same reaction conditions. After the first polymerisation, the wire was kept wrapped around the stirring bar, washed in acetone, dried under nitrogen flow and stored in a nitrogen atmosphere until the next reaction. The stirrer with the copper wire was washed with methanol and dried under nitrogen right before starting a new experiment.

**Fig. 4:**
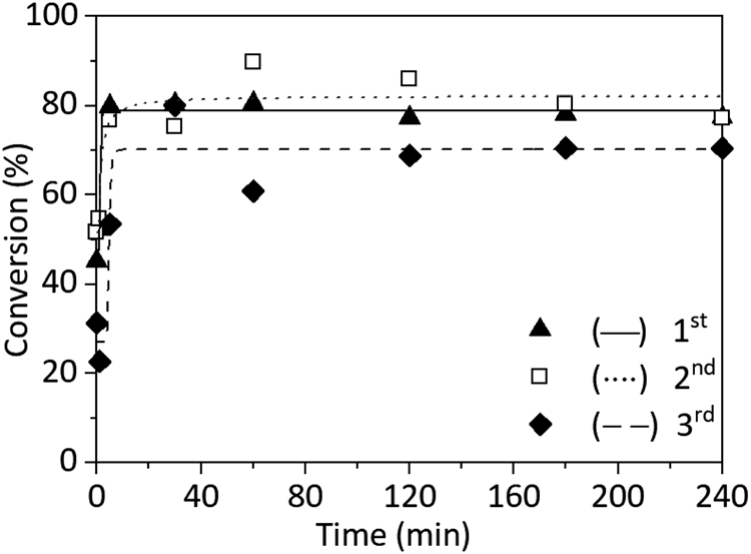
Conversion plots of SET-RDRP of NAGA in water (0.75 M), at 25 °C, under a nitrogen blanket, with catalytic system [NAGA]_0_ : [CPA]_0_ : [Me_6_TREN]_0_ : [Cu^II^]_0_ = 500 : 1 : 0.2 : 0.1, and catalysis of copper wire (18 gauge, 4 cm). The same copper wire was used for all three polymerizations. Data were fitted with a 5PL fit.

All three SET-RDRP of NAGA exhibited similar kinetics (Fig. 4 and ESI Table S2†). Both during the first and second SET-RDRP after 5 min, the conversion was already around 80%. This conversion was reached after 30 min during the third polymerisation. After 4 h, the theoretical molecular weights correlated well with the values obtained from SEC and the *Đ* was between 1.84 and 1.98. The molecular weight distributions of each polymerisation are completely overlapping (Fig. S13†). Therefore, using a copper wire for the catalysis would reduce the need for pristine metal, reduce cost and avoid metal contamination of the product.

### Phase transition performance of synthesized PNAGA

**Fig. 5:**
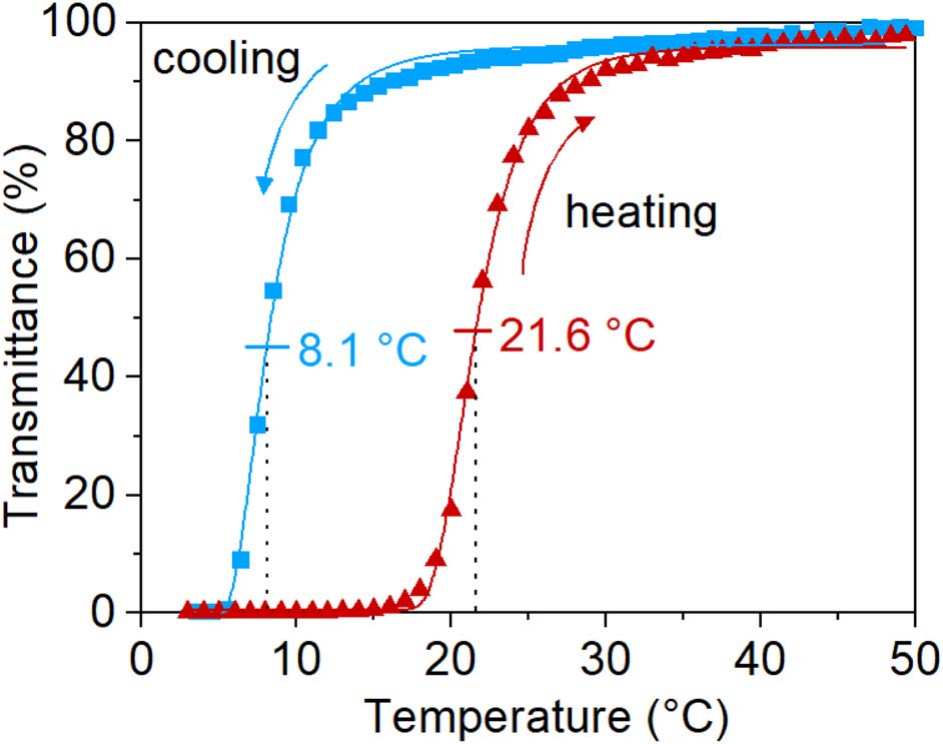
Turbidimetry curve of PNAGA (18 700 g mol^−1^, *Đ* = 3.1, from the kinetic study in Fig. 1e, f) in deionized water. The data were fitted with 5PL fit and the cloud points were determined from the inflection points.

As mentioned in the introduction, the UCST-properties of PNAGA are highly affected by the choice of reaction parameters. The UCST phase transition properties of the newly synthesized polymers *via* SET-RDRP were confirmed on UV-vis by observing the change in transmittance with temperature. To prevent the hydrolysis of the polymer side chains during sample preparation, a poly(propylene) container was used to dissolve the polymer in deionized water at 60 °C.^13^ PNAGA prepared *via* SET-RDRP preserved the temperature-responsiveness. The sharp UCST phase transition can be observed in Fig. 5. It exhibited a cloud point of 8.1 °C upon cooling and 21.6 °C upon heating. Three consecutive cycles of the turbidimetry measurement can be observed in Fig. S14.†

## Conclusions

We have presented the synthetic procedure of PNAGA *via* SET-RDRP. Polymerisation of PNAGA with a targeted DP_*n*_ of 500 was achieved in water at ambient temperatures and non-inert conditions with a copper wire as a catalyst. The reusability of the catalyst up to three times is demonstrated. Furthermore, the proposed synthesis of PNAGA was successfully carried out in a simple vial without any tedious reaction preparations. Temperature-responsive polymers have been of interest, especially in biomedicine^4,55^ where materials of high purity are required. Herein, the reduction of the metal content in the reaction medium together with the simple removal of the catalyst facilitates the purification of the material.

## Conflicts of interest

The authors declare no conflicts of interest.

## Supplementary Material

RA-013-D3RA04993K-s001
