# Supplementary material for: Copper-mediated synthesis of temperature-responsive poly(N-acryloyl glycinamide) polymers: a step towards greener and simple polymerisation
Source: RSC Adv. 2023 Oct 4;13(42):29099–108. doi: 10.1039/d3ra04993k (PMC10548432; doi:10.1039/d3ra04993k)
Supplement: RA-013-D3RA04993K-s001 [file RA-013-D3RA04993K-s001.pdf]

Electronic Supplementary Material (ESI) for RSC Advances.
This journal is © The Royal Society of Chemistry 2023

Supplementary information

Copper-mediated synthesis of temperature-responsive poly(*N*-acryloyl glycinamide) polymers: A step towards greener and simple polymerisation

Nikola Křivánková,[*a, c] Kerem Kaya,[b, c] Wouter van der Wijngaart[b, c] and Ulrica Edlund[a, c]*

[a] Fibre and Polymer Technology, School of Engineering Sciences in Chemistry, Biotechnology and Health, Royal Institute of Technology (KTH), Stockholm 100 44, Sweden

[b] Intelligent Systems, School of Electrical Engineering and Computer Science, Royal Institute of Technology (KTH), Stockholm 100 44, Sweden

[c] Digital Futures, Royal Institute of Technology (KTH), Stockholm 100 44, Sweden

N-acryloyl glycinamide (NAGA) monomer

The ^{1}H NMR spectrum of the NAGA monomer with assigned protons to peaks is shown in Figure S1. An example of the obtained DSC curve with labelled melting temperature is shown in Figure S2. After melting, NAGA undergoes polymerization indicated by the exothermic heat flow.

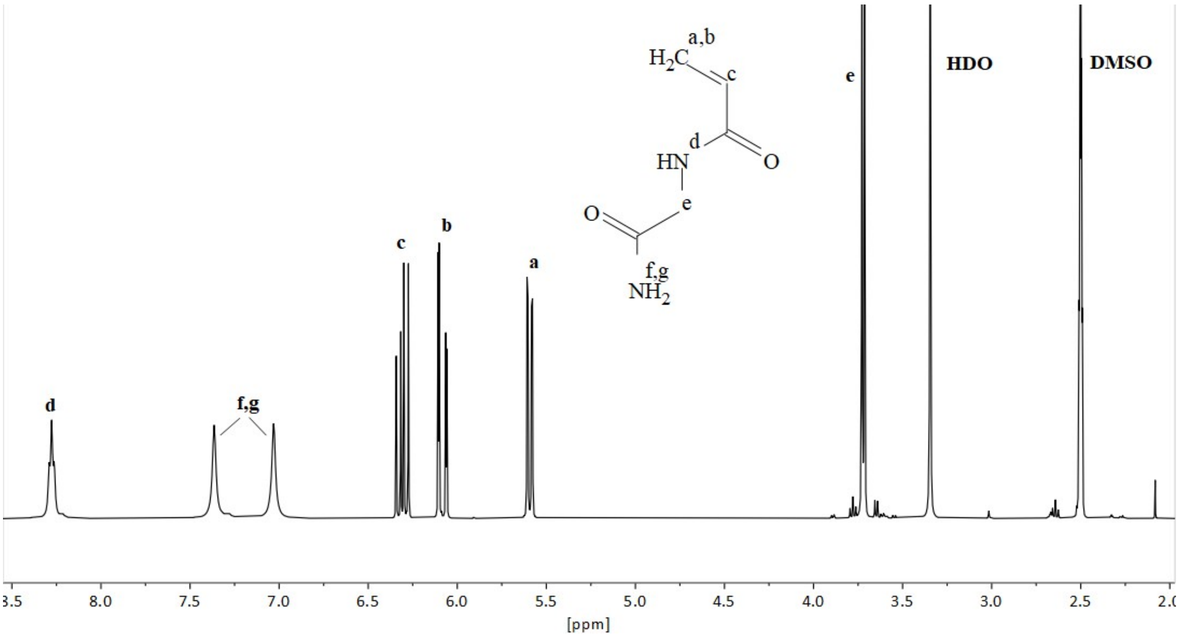

Figure S1: ^{1}H NMR spectrum of NAGA monomer in DMSO-d_6 at room temperature.

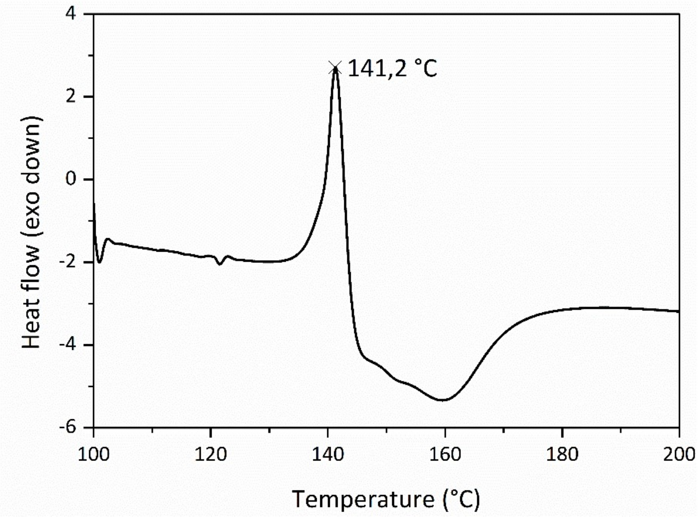

Figure S2: An example of the synthesized monomer NAGA DSC curve with highlighted melting temperature.

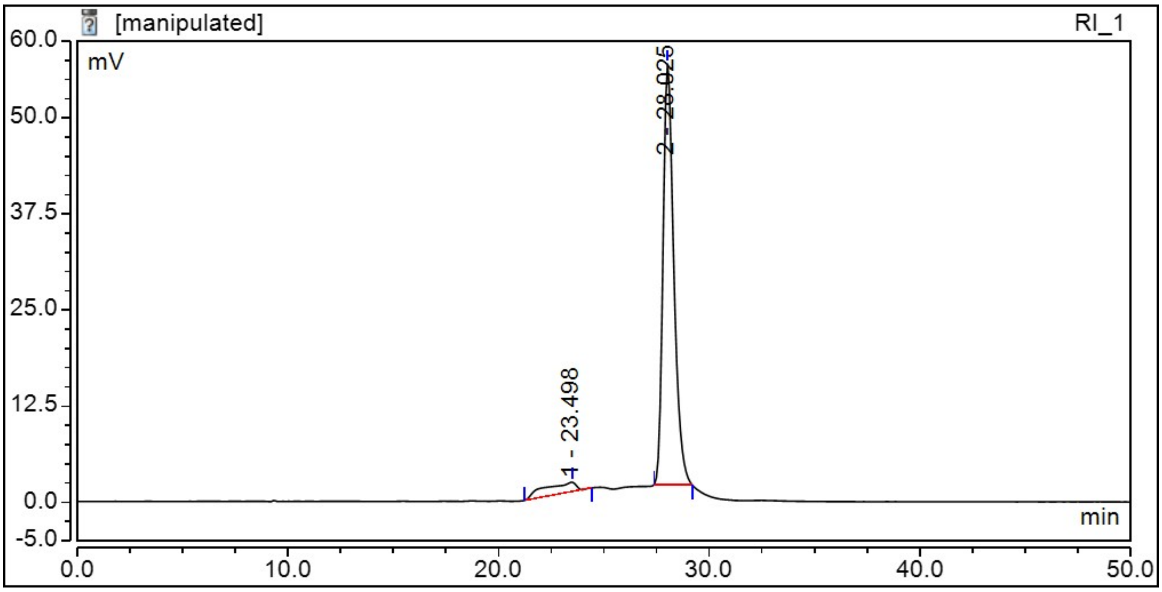

Figure S3: HPLC elugram of the NAGA monomer. By comparing the peak areas of the monomer and the impurities, the monomer purity was determined to be 93 %.

Further characterization of poly(*N*-acryloyl glycinamide)

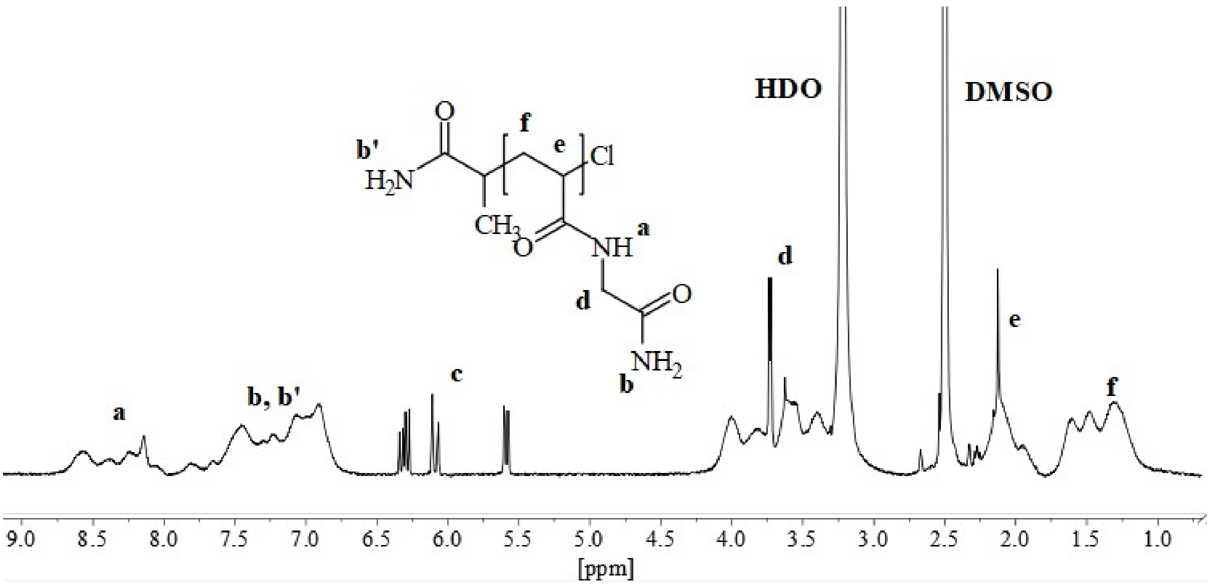

Figure S4: ¹H NMR spectrum with assigned peaks to an impurified PNAGA polymer in DMSO-d_6 at 50 °C. Peaks between 6.5 and 5.5 ppm belong to the vinyl protons of an unreacted monomer.

Explanation of processing of kinetics data

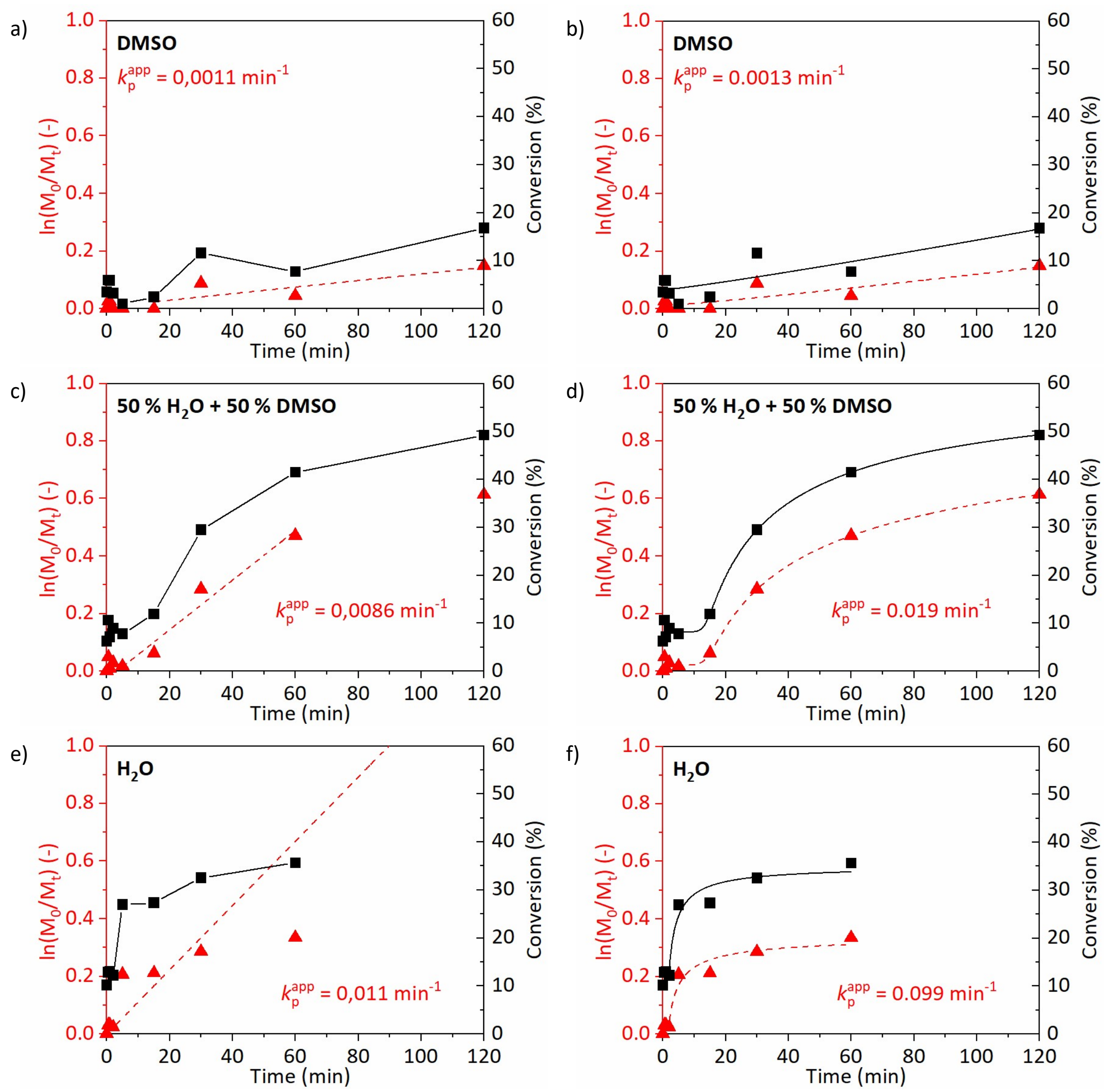

Figure S5: Comparison of different data processing. Left: Black connecting lines are for eye guidance only. Red dashed lines are linear regressions for the determination of k_p^{app} (a, c, e). Right: Data were plotted with a five-parameter logistic fit (Eq. 1). k_p^{app} was calculated as the maximum of the first derivative of the five-parameter logistic fit curve (b, d, f).

Molecular weight distributions

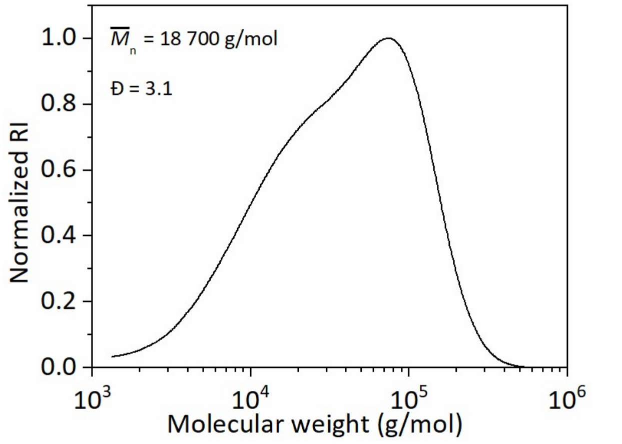

Figure S6: Molecular weight distribution of PNAGA after 60 min while polymerized at 45 °C, in deoxygenated water (1.5 M), under the conditions $[NAGA]_0$:$[CPA]_0$:$[Me_6TREN]_0$:$[Cu^{II}]_0$ = 500:1:2:1 and catalysis of a copper wire (6 cm, gauge 18).

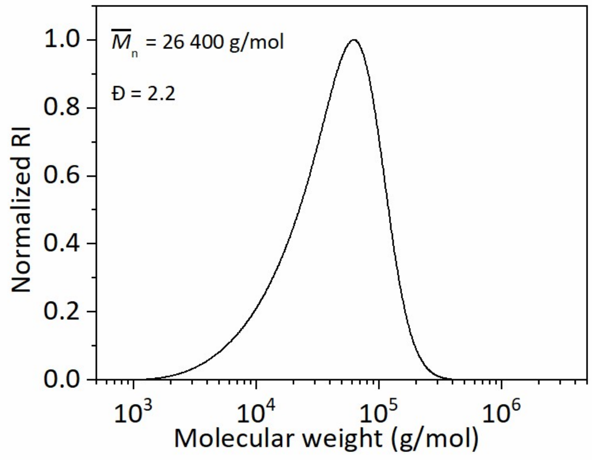

Figure S7: Molecular weight distribution of PNAGA after 120 min while polymerized at 25 °C, in deoxygenated water (1.5 M), under the conditions $[NAGA]_0$:$[CPA]_0$:$[Me_6TREN]_0$:$[Cu^{II}]_0$ = 500:1:2:1 and catalysis of a copper wire (6 cm, gauge 18).

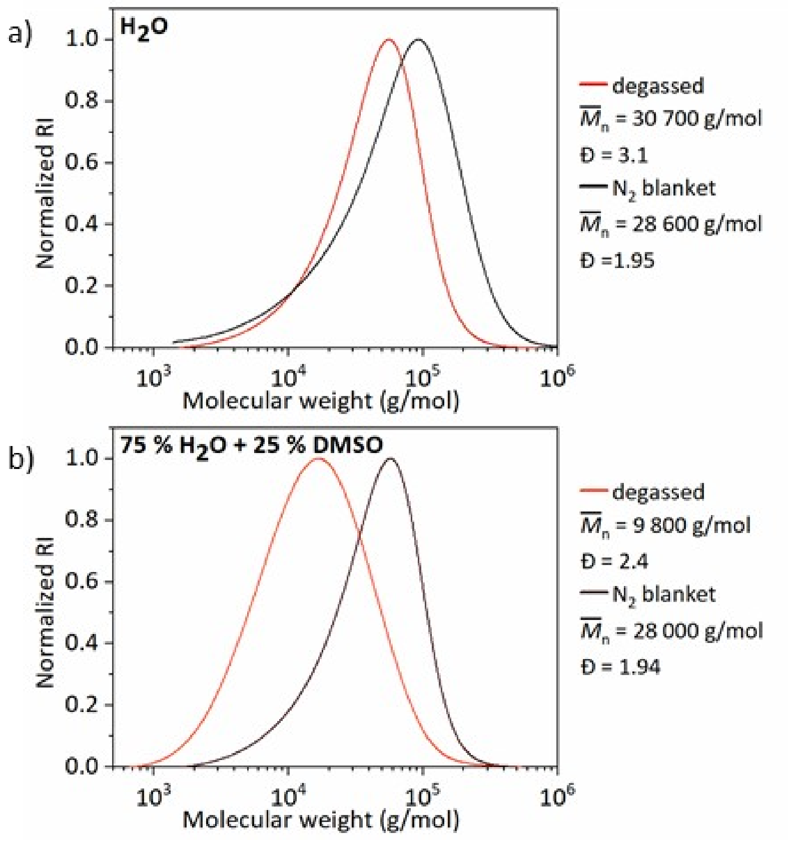

Figure S8: Molecular weight distributions of PNAGA when synthesized in a deoxygenated (red) and non-deoxygenated (black) conditions either in water (a) after 60 min and 75% water and 25% DMSO solution (b) after 180 min. Reaction conditions were 25 °C, monomer concentration 1.5 M, $[NAGA]_0$:$[CPA]_0$:$[Me_6TREN]_0$:$[Cu^{II}]_0$ = 500:1:2:1 and catalysis with a copper wire (6 cm, gauge 18).

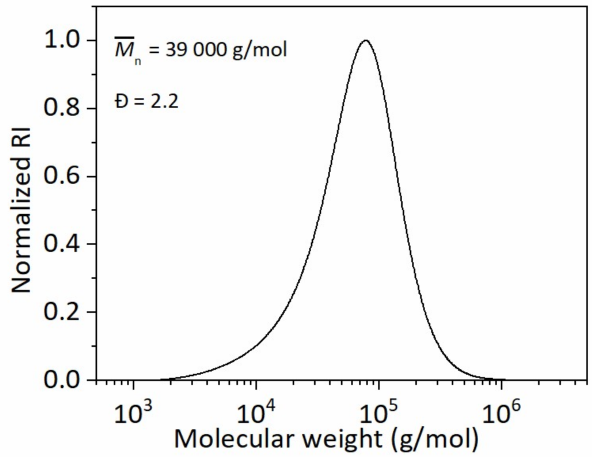

Figure S9: Molecular weight distribution of PNAGA polymerized in a closed vial at 25 °C, in non-deoxygenated water (0.75 M), under the conditions $[NAGA]_0$:$[CPA]_0$:$[Me_6TREN]_0$:$[Cu^{II}]_0$ = 500:1:0.2:0.1 and catalysis of a copper wire (6 cm, gauge 18).

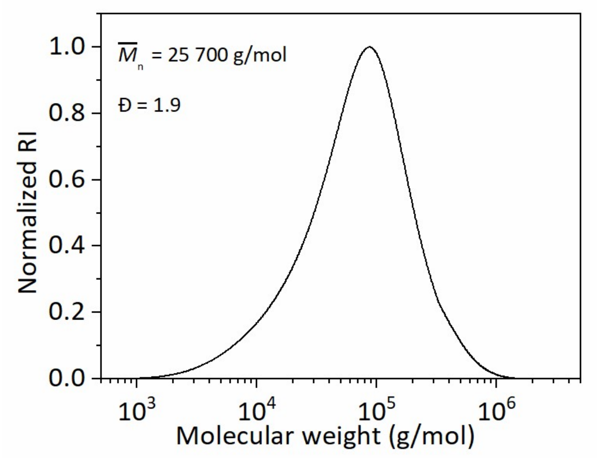

Figure S10: Molecular weight distribution of PNAGA after 240 min while polymerized at 25 °C, in non-deoxygenated water (1.5 M), under the conditions $[NAGA]_0:[CPA]_0:[Me_6TREN]_0:[Cu^{II}]_0$ = 500:1:4:2 and catalysis of a copper wire (6 cm, gauge 18).

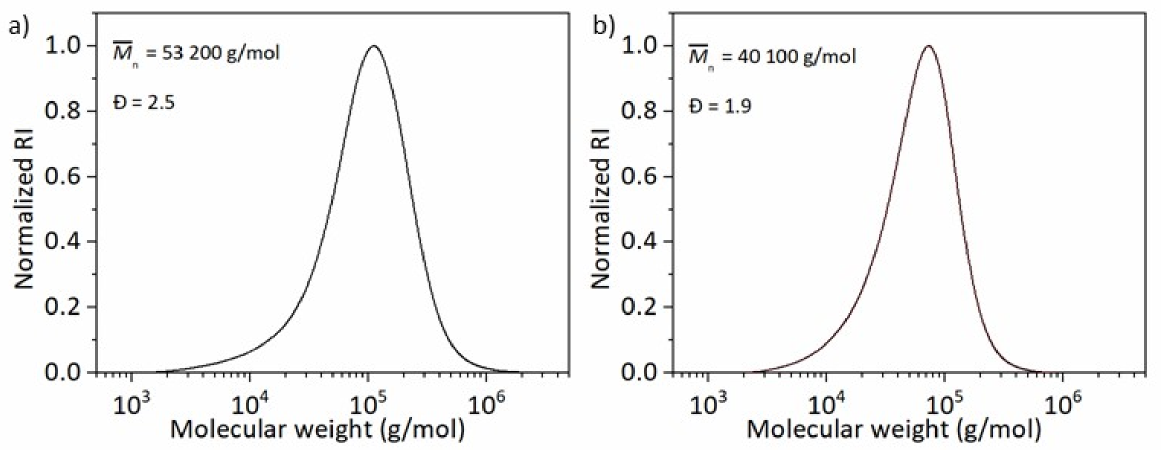

Figure S11: Molecular weight distribution of PNAGA after 60 min while polymerized at 25 °C, in non-deoxygenated water, under the conditions $[NAGA]_0:[CPA]_0:[Me_6TREN]_0:[Cu^{II}]_0$ = 500:1:0.2:0.1 and catalysis of a copper wire (6 cm, gauge 18). Initial monomer concentration was 1.5 M (a) and 0.75 M (b).

In situ **chain extension**

Table S1: The theoretical and calculated degrees of polymerisation of the samples showing the presence of living chain ends.

T [°C]	$DP_{n,theo}$	$DP_{n,real}$	$M_{n,theo}$ [g/mol]	$M_{n,SEC}$ [g/mol]	Đ
25	50	40.5	6 200	11 200	1.5
	100	74.9	12 000	10 800	1.2
45	50	32.2	6 400	8 200	1.2
	100	65.4	12 300	11 600	1.7

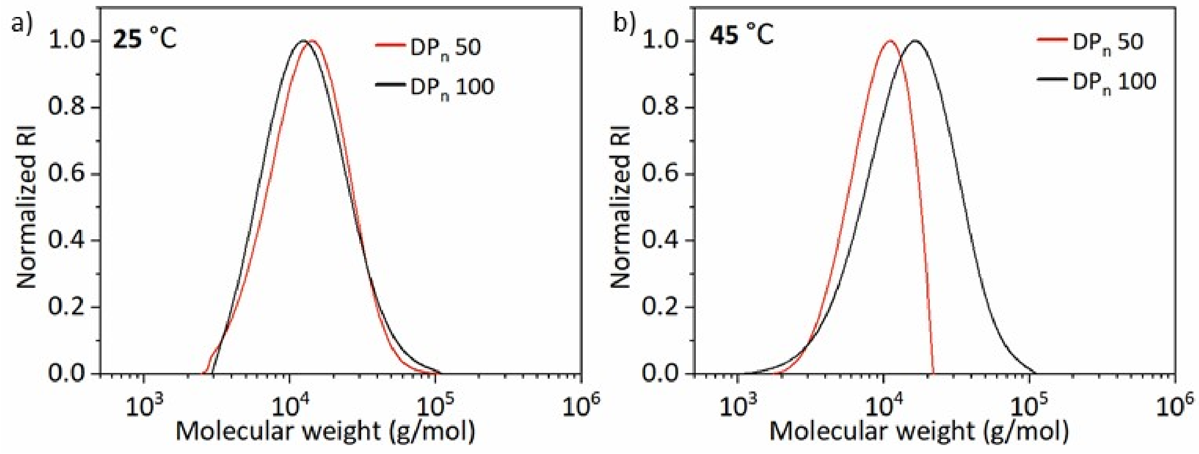

Figure S12: Molecular weight distribution of PNAGA with theoretical DP_n 50 and after chain extension to DP_n 100 proceeded either at 25 (a) or 45 °C (b) in deoxygenated water (1.5 M) under the conditions $[NAGA]_0:[CPA]_0:[Me_6TREN]_0:[Cu^{II}]_0$ = 50:1:2:1 and catalysis of a copper wire (6 cm, gauge 18).

Wire recyclability

Table S2: Conversions, molecular weights, and polydispersities of three cycles of SET-RDRP of NAGA in water (0.75 M), at 25 °C, under a nitrogen blanket, with catalytic system $[NAGA]_0:[CPA]_0:[Me_6TREN]_0:[Cu^{II}]_0$ = 500:1:0.2:0.1, and catalysis of copper wire (18 gauge, 4 cm). The same copper wire was used for all three polymerizations.

Rep.[a]	Time [min]	Conv. [%]	$M_{n,theo}$ [g/mol]	$M_{n,SEC}$ [g/mol]	Đ
1	1	52	33 100	59 800	2.09
	5	80	50 500	52 600	2.10
	30	80	50 800	43 900	1.92
	240	77	49 000	47 800	1.93
2	1	54	34 600	51 600	1.94
	5	76	48 600	56 200	1.99
	30	75	47 700	65 200	2.32
	240	77	48 900	47 900	1.84
3	1	23	14 400	43 100	1.79
	5	53	33 900	34 500	1.80
	30	80	50 700	55 300	1.82
	240	70	44 600	53 600	1.98

[a] the number of repetitions

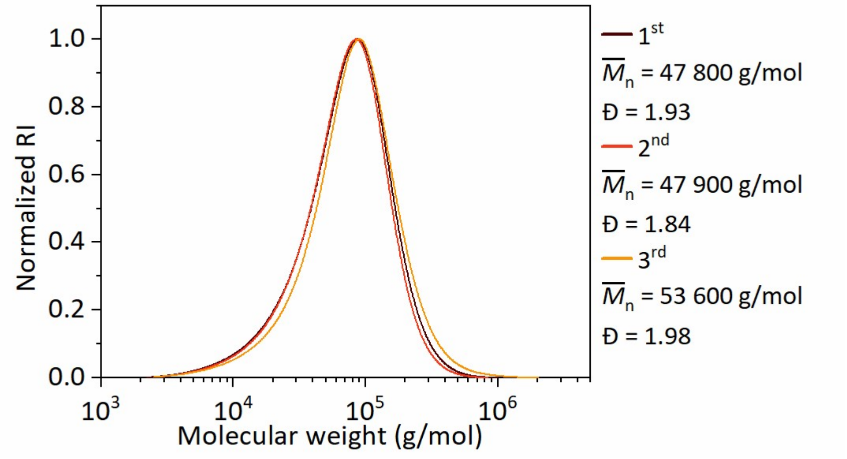

Figure S13: Molecular weight distributions of PNAGA polymerized with catalysis of the same copper wire (18 gauge, 4 cm). The wire was used for three polymerisations. The reaction conditions were the following: water (0.75 M), 25 °C, under a nitrogen blanket, with a catalytic system $[NAGA]_0$:$[CPA]_0$:$[Me_6TREN]_0$:$[Cu^{II}]_0$ = 500:1:0.2:0.1.

Temperature-responsiveness of PNAGA

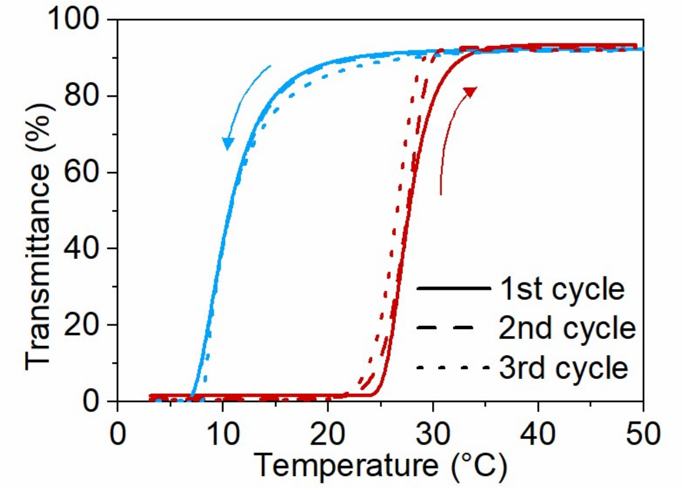

Figure S14: Turbidimetry curves of three consecutive measurements of PNAGA with $\bar{M}_n$ = 17 600 g/mol, Đ = 2.1 in water (1 wt.%). The lines represent five-parameter logistic fits of each set of data.
